# What imaging modalities should be considered in suspected acute acalculous cholecystitis? A review of the evidence

**DOI:** 10.1186/s13244-025-02106-2

**Published:** 2025-11-19

**Authors:** Benjamin Simon Phipps, Helen Kavnoudias, Bruno Di Muzio

**Affiliations:** 1 Department of Radiology, Alfred Hospital, Melbourne, VIC Australia; 2 Department of Neuroscience and Surgery, Monash University, Melbourne, VIC Australia

**Keywords:** Acalculous cholecystitis, Gallbladder, Nuclear medicine, Ultrasonography, Magnetic resonance imaging

## Abstract

**Background:**

Radiological assessment remains crucial for acute acalculous cholecystitis (AAC) diagnosis, however, there is debate regarding the optimal imaging pathway. In the clinical setting, the decision to intervene, and the chosen procedure, are greatly influenced by imaging findings, and there is a need for a clear evaluation of each imaging modality’s proficiency for AAC detection and its prognostic utility.

**Methods:**

We performed a survey of the literature on the radiological diagnosis of AAC. Prospective and retrospective studies were selected if they examined the diagnostic utility of the imaging modality using histology as the ground truth, and had a sample size of greater than ten patients.

**Results:**

Seventeen relevant studies were identified, which analysed US, hepatobiliary iminodiacetic acid (HIDA) scan, CT or MRI. The US has a reported specificity of between 93% and 97%, however, the sensitivity varied widely from 20% to 100%. The specificity of HIDA was reported as between 78% and 100%, but again, the sensitivity varied, between 38% and 100%. The literature on CT and MRI is limited, and there is no clear benefit over US for AAC diagnosis, however, they may be valuable for ruling out other diagnoses, or for surgical planning.

**Conclusion:**

While radiological assessment holds utility in the management of suspected AAC, further research is required to properly define its role. Future research should focus on well-designed prospective studies to establish the diagnostic performance of both individual and multimodal imaging strategies in AAC, as well as the standardisation of imaging criteria and protocols across institutions.

**Critical relevance statement:**

Prompt radiological diagnosis of AAC can prevent complications and improve patient survival, however, radiological assessment still varies between institutions. Consequently, there is an urgent need for the diagnostic performance of imaging strategies to be established, and protocols across institutions to be standardised.

**Key Points:**

- Radiological assessment is crucial for early diagnosis and prompt management of AAC.
- CT, MRI and HIDA scans demonstrate minimal benefit over US for diagnosis.
- Further research is required for evidence-based standardisation of imaging criteria and protocols.

**Graphical Abstract:**

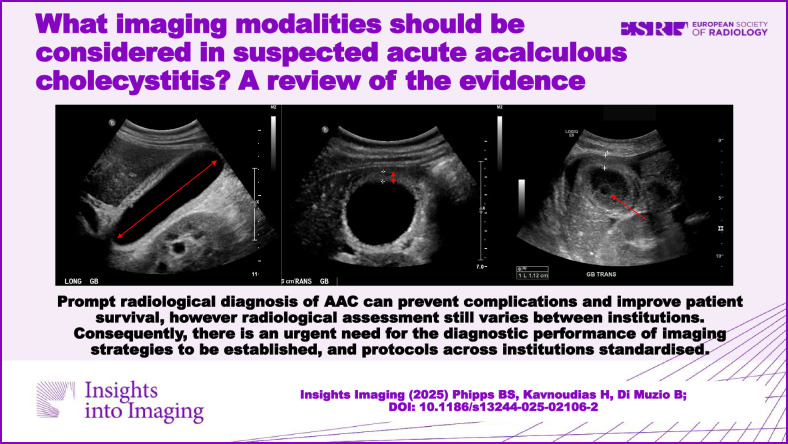

## Introduction

### The unclear pathogenesis of acute acalculous cholecystitis (AAC)

AAC was first described over 150 years ago by Dr Duncan, yet despite its long history, it remains one of the most poorly understood conditions in modern medicine [1]. It is defined simply as inflammation of the gallbladder in the absence of stones, however, the physiological mechanisms driving this inflammation are complex, and researchers cite several potential causes [2, 3]. Histologically, there are similarities between AAC and acute calculous cholecystitis (ACC), in both cases, inflammatory cell infiltration and epithelial degeneration are common in early disease, whereas adipose tissue and muscle layer necrosis are observed in more advanced disease [4]. However, while in ACC this is caused by mechanical irritation, wall oedema, and vessel occlusion as a consequence of outlet obstruction by calculi, the aetiology in AAC is less clear [5, 6].

Gallbladder stasis, sometimes referred to as gallbladder ileus or dyskinesia, plays an important role in the development of AAC. Studies of gallbladder smooth muscle cells from AAC patients demonstrate reduced spontaneous contractility and an impaired response to contractile agonists, and show increased bile infiltration of the mucosal smooth muscle layers from prolonged biliary stasis [3, 7, 8]. It is theorised that this leads to the inflammation seen in AAC by preventing the elimination of sediment and crystals, resulting in secondary injuries to the gallbladder wall, while providing an ideal environment for bacterial proliferation [9].

Gallbladder infection, systemic inflammation and gallbladder ischaemia are additionally implicated in the development of AAC. Gallbladder infection appears to precipitate both biliary inflammation and stasis. The hydrolysis of bile salts into bile acid from biliary bacterial pathogens causes a rise in serum lipid, which subsequently has a toxic hydrophobic effect on the gallbladder, interfering with smooth muscle function to cause biliary stasis [9, 10]. Further, biliary infection raises histamine levels, which act to increase cystic duct resistance, worsening stasis [9, 11]. Alternatively, injury from systemic inflammation and ischaemia has been proposed as the primary drivers, particularly given AAC occurs in the absence of gallbladder infection in over half of all cases [12–14]. In evidence of this, a decrease in the cytoplasmic expression of occludin and claudin-1, both of which are downregulated during a global inflammatory response, is characteristic of AAC pathology specimens [15]. In addition, AAC specimens demonstrate an increase in apoptosis, cellular proliferation, and leucocyte margination in blood vessels, all signs of ischaemia reperfusion injury [3, 14]. Ischaemia from microthrombi is also implicated, and histopathological studies have identified capillary thromboses and irregular filling on microangiopathy of AAC specimens [3, 16]. Finally, the key risk factors associated with the development of AAC are impaired circulation (shock time, catecholamine requirement), ventilation requirement (duration of ventilator use), and inflammation (raised white cell count (WCC), raised C-reactive protein (CRP)), which aligns closely with the theorised causes of AAC being ischaemia and infection (Table 1).

**Table 1:** Clinical variables associated with the development of AAC

Clinical factors	Reported *p* value
Associated with AAC in the ICU patient cohort	
Older age [25]	< 0.001
Increased shock time [79]	< 0.001
Increased catecholamine requirement [25, 79]	< 0.001
Antibiotic requirement [25]	< 0.001
Increased blood transfusion requirement [25, 41]	< 0.001
Inhalation injury [25]	< 0.001
Increased duration of ventilator use [25, 79]	< 0.001
Admitted on ventilator [25]	0.004
Increased duration of sedation [79]	< 0.001
Longer length of stay [25]	< 0.001
Raised WCC [79]	< 0.001
Raised CRP [79]	< 0.001
Increased heart rate [41]	0.007
Higher APACHE-II score [41, 79]	< 0.001
Associated with AAC in patients with cholecystitis	
Older age [26]	0.004
Male sex [27]	0.049
COPD [26]	< 0.001
No history of biliary colic [26]	0.024
Fever at presentation [26]	< 0.001
Hypotensive at presentation [26]	< 0.001
Albumin < 30 g/L [26]	0.019
Higher Charlson comorbidity index [27]	0.004
Acute cholangitis [27]	0.007

*AAC* acute acalculous cholecystitis, *APACHE-II* acute physiology and chronic health evaluation II, *COPD* chronic obstructive pulmonary disease, *CRP* Creactive protein, *ICU* intensive care unit, *WCC* white cell count

Additional theories and proven mechanisms for the aetiology of AAC exist. Physical obstruction of the common bile duct due to a mass-lesion (e.g. metastatic disease) can cause distention and oedema of the gallbladder, leading to hypoperfusion and eventually necrosis. Rarely, gallbladder torsion, a condition where the gallbladder twists along its axis, can present as AAC, mimicking its clinical features. In addition, hyperlipidaemia has been implicated—raised cholesterol levels increase the epithelial permeability and reduce the contractility of the gallbladder, resulting in inflammation and stasis, both important mediators of AAC [17].

### Why does AAC diagnosis pose a clinical dilemma?

It is widely recognised that AAC occurs commonly in the critically ill, likely because patients have many predisposing factors for gallbladder stasis, including fasting, parental nutrition use, and post-surgical ileus, as well as being more likely to experience risk factors such as shock, ventilation requirement, and thrombi. In the intensive care unit (ICU) cohort, the incidence is less than 5% [4, 18–22], but is much higher when these patients have a clinical picture suggestive of cholecystitis, between 32% and 50% [18–20]. Diagnosing AAC in this cohort is challenging, as traditional clinical assessment through history and examination is often not possible. Moreover, these patients present with nonspecific symptoms such as right upper quadrant pain, fever, leucocytosis, and deranged liver function tests (LFTs) [2, 23]. As a consequence, clinicians will rely heavily on imaging for diagnosis. Unfortunately, however, the rarity of the condition, clinical complexity of the patient base, and the absence of consistent imaging diagnostic criteria make identifying AAC with imaging a significant challenge.

Nevertheless, the importance of rapid diagnosis and treatment of AAC is clear when examining patient outcomes. The dreaded complications of AAC—gangrene, abscess formation, and perforation—are relatively common and carry significant mortality risk if not appropriately managed [24]. The incidence of gangrene in AAC is reported between 16.1% and 63% [25–29], wall abscess between 3% and 6% [25, 27, 28], and perforation between 5% and 20% [20, 25, 27, 28]. In terms of absolute mortality, the risk is cited as between 0% and 12% [24, 26, 27], and between 43.5% and 53.3% in patients who are critically ill [4, 25, 28]. Evidently, AAC carries a high morbidity and mortality, and early radiological detection and intervention are paramount. In this paper, we explore the available imaging modalities for AAC diagnosis and compare their utility for the detection, management, and prognostication of the disease.

## Scoping review rationale and aim

As is clearly outlined in the 2018 Tokyo Guidelines for Management of Acute Cholangitis and Acute Cholecystitis (TG18), imaging is a requirement for acute cholecystitis diagnosis, in combination with clinical features and associated systemic signs of inflammation [30]. However, the primary imaging modality and radiological diagnostic criteria for AAC are not stated, and substantial debate remains around the subject. Ultrasound (US) is the most recognised modality, yet its reported utility varies significantly, and research indicates it is prone to false positives [21, 22]. Hepatobiliary iminodiacetic acid (HIDA) scans are recommended by some institutions if a US result is equivocal, but the benefit is not clearly defined, and the lengthy acquisition process can be risky for critically unwell patients, raising concern around its use in this context [2, 19]. Similarly, the role of computed tomography (CT) and magnetic resonance imaging (MRI) is not clearly described [13, 31].

Given that the management of AAC usually requires surgical or interventional radiology action, which carries its own risks, an accurate imaging diagnosis is crucial. In the clinical setting, the decision to intervene, and the chosen procedure, are greatly influenced by imaging findings, and there is a need for a clear evaluation of both the imaging modality’s proficiency for AAC detection and its prognostic utility. This scoping review aims to address this need by evaluating the diagnostic performance, strengths, and limitations of the key imaging modalities in AAC, providing insight into their role in clinical decision-making.

## Scoping review methods

The literature search was conducted in the PubMed and OVID Medline electronic databases. A comprehensive search strategy designed to capture all published research on an imaging diagnosis of AAC was performed. Terms such as “acalculous” and “cholecystitis” were used, as well as keywords related to imaging modalities, such as “ultrasound,” “computed tomography,” “magnetic resonance imaging,” “hepatobiliary scintigraphy,” and “HIDA scan,” to encompass all relevant diagnostic approaches. To enhance the sensitivity of the search, the reference lists of included articles were manually screened for additional relevant studies. Given the significant heterogeneity in study designs, methodologies, and definitions of AAC, a narrative synthesis of results was chosen. The preferred reporting items for systematic reviews and meta-analyses extension for scoping reviews (PRISMA-ScR) guidelines were used to identify relevant studies [32]. Further details on methods, including a full search strategy, are provided in the supplementary materials.

## Scoping review results

Seventeen relevant studies were identified, and are outlined in Tables 2–4. Eleven studies tested the efficacy of US, 9 examined HIDA scans, and 3 examined CT (some studies tested multiple modalities). No studies were found that tested the utility of MRI for AAC diagnosis specifically. The following sections present the findings for each modality.

**Table 2:** Comparison of the literature on sonographic diagnosis of AAC

Author	No. of patients	Demographic	US diagnostic criteria	Sensitivity	Specificity	Study type
Becker et al [35]	11	Previous major surgery during admission	Wall thickness ≥ 4 mm	90.9%	-	Case series
Beckman et al [33]	16	Inpatient cholecystectomy	Wall thickness ≥ 3 mm if gallbladder distended, ≥ 5 mm if not	81%	-	Case series
Blankenberg et al [37]	17	Inpatient cholecystectomy	Two major or, one major and two minor^1^	46%	-	Case series
Shuman et al [42]	22	Inpatient cholecystectomy	Wall thickening greater than 6 mm, gallbladder distension, or echogenicity in the gallbladder lumen suggestive of pus, in combination with a sonographic Murphy sign.	63%	-	Case series
Mariat et al [19]	28	ICU	3 major signs (sludge, distension (10 × 4 cm), wall thickening (≥ 4 mm)	50%	94%	Cohort study
Prévôt et al [18]	32	ICU	Three major signs (sludge, distension (10 × 4 cm), wall thickening (≥ 4 mm)	36%	89%	Cohort study
Mirvis et al [43]	60	Trauma	Two major or one major + 2 minor^1^	92%	96%	Cohort study
Deitch et al [40]	5 (with 60 healthy controls)	Inpatient cholecystectomy	Wall thickness ≥ 3 mm	100%	90%	Case control study (healthy controls)
Puc et al [20]	66	Trauma	Wall thickening (> 3.5 mm) or pericholecystic fluid collection, or emphysematous gallbladder.	30%	93%	Cohort study
Pelinka et al [41]	255	Trauma	Thickening of the gallbladder wall (≥ 3.5 mm), additional layering or necrotic degeneration, oedema of the surrounding tissue, and/or impending rupture.	100%	97.4	Cohort study
Hill et al [80]	8	Inpatient cholecystectomy	One of either pericholecystic fluid, wall thickening or intramural air.	75%	-	Case series

For the ICU and Trauma cohort studies, the patients scanned had a clinical picture that was suggestive of cholecystitis

^1^ Major criteria include: thickened gallbladder wall (> 4 mm), pericholecystic fluid, sub-serosal oedema, intramural gas, sloughed mucosa, pericholecystic fat infiltration, complete lack of response to cholecystokinin, and a positive sonographic Murphy’s sign. Minor criteria include: presence of sludge, distention of the gallbladder (> 8 cm in the longitudinal or > 5 cm in the transverse dimension), and partial response to cholecystokinin

**Table 3:** Comparison of the literature on HIDA diagnosis of AAC

Authors	No. of patients	Demographic	HIDA diagnostic criteria	Sensitivity	Specificity	Study type
Kalliafas et al [28]	10	Inpatient cholecystectomy	MC—wait 90 min, if not visualised, morphine sulphate was injected	90%	N/A	Case study
Ramanna et al [53]	11	Inpatient cholecystectomy	Non-visualisation of the gallbladder	100%	N/A	Case study
Shuman et al [42]	12	Inpatient cholecystectomy	Non-visualisation of the gallbladder for 4 h	68%	N/A	Case study
Weissmann et al [54]	15	Inpatient cholecystectomy	Non-visualisation of the gallbladder for up to 3 h	93.30%	N/A	Case study
Puc et al [20]	20	ICU	Non-visualisation of the gallbladder (time period not specified)	100%	88%	Cohort study
Mariat et al [19]	31	ICU	Non-visualisation of the gallbladder for 60 min + an additional 60 min after morphine administration	67%	100%	Cohort study
Prévôt et al [18]	32	ICU	Non-visualisation of the gallbladder for 60 min + an additional 30 min after morphine administration	MC: 64% RC: 86%	MC: 100% RC: 78%	Cohort study
Swayne et al [55]	40	Inpatient cholecystectomy	Non-visualisation of the gallbladder for at least 2 h OR gallbladder perforation visualised	95%	N/A	Case study
Mirvis et al [43]	46	Trauma	Non-visualisation of the gallbladder for up to 6 h	38%	95%	Cohort study

*MC* morphine cholescintigraphy, *RC* radionuclide cholescintigraphy

**Table 4:** Comparison of the literature on the CT diagnosis of AAC

Authors	No. of patients	Demographic	CT diagnostic criteria	Sensitivity	Specificity	Study type
Ahvenjarvi et al [31]	127	Inpatient cholecystectomy	Standard diagnostic criteria^1^	22%	NA	Case-control study
Mirvis et al [43]	15	Trauma	Standard diagnostic criteria^1^	100%	100%	Cohort study
Blankenberg et al [37]	10	Inpatient cholecystectomy	Standard diagnostic criteria^1^	50%	NA	Case study

For the cohort studies, the patients scanned had a clinical picture that was suggestive of cholecystitis

^1^ The presence of two major or two minor and one major finding is used to make a diagnosis. Major criteria include: wall thickening greater than 4 mm, pericholecystic fluid, subserosa oedema without ascities, intramural gas, and sloughed mucosa. Minor criteria include: Subjective distension and hyperdense bile [43]

### Ultrasound

**Fig. 1:**
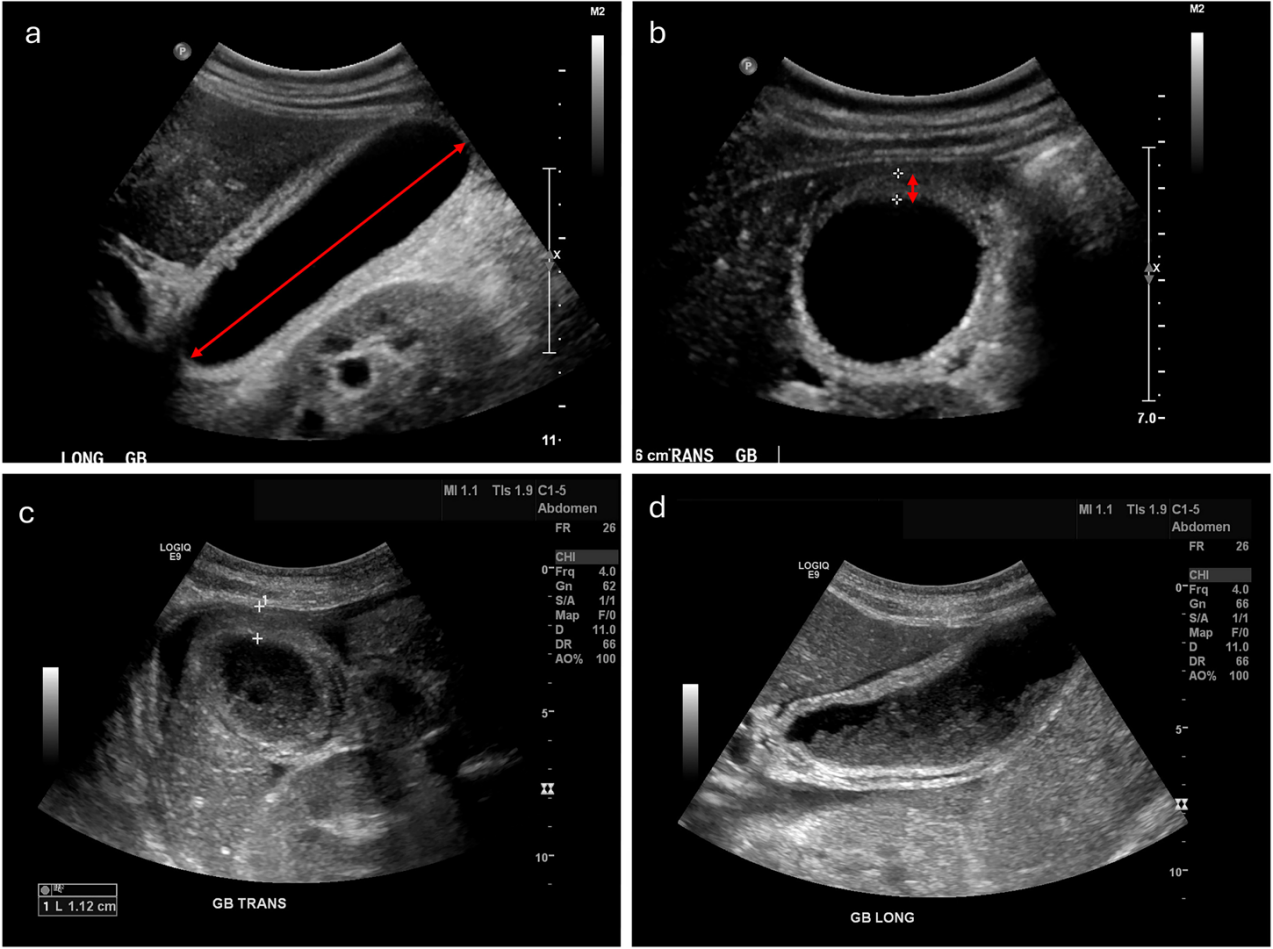
US findings in confirmed cases of acalculous cholecystitis demonstrate variability in imaging appearances. Case 1 **a**, **b**: longitudinal view (**a**) shows a markedly distended gallbladder (longitudinal axis length > 8 cm) with anechoic fluid content consistent with hydrops; transverse view (**b**) demonstrates wall thickening > 3 mm supporting inflammation. Case 2 (**c**, **d**): gallbladder distension with a significantly thickened oedematous wall (11 mm), heterogeneous biliary sludge, and pericholecystic fluid

US is considered the primary diagnostic modality for AAC, and a key reason for this is its mobility. Given that the patient population who present with AAC are often critically ill and immobilised, this alone makes it preferable to the other modalities. When looking at the diagnostic criteria for AAC, significant variation is evident within the literature. Signs include gallbladder wall thickening (> 3 mm), pericholecystic fluid or subserosal oedema, intramural gas, sloughed mucosal membrane, gallbladder distension, and sonographic Murphy’s sign (Fig. 1) [30, 33–37]. Important differentials include ACC, where gallstones are visible on US, and ascending cholangitis, which would demonstrate thickening of the bile duct wall secondary to obstruction [38, 39]. The following section examines the diagnostic and prognostic utility of US for AAC (Table 2).

#### Diagnostic utility of US in AAC

Dietch et al’s research was the first to define a sonographic criterion for the diagnosis of AAC, demonstrating a gallbladder wall thickness of 3 mm or greater to have a specificity of 90% and a sensitivity of 100% for AAC, and at present, this criterion remains the gold standard for diagnosis [40]. A key issue with this study is the use of healthy patient controls, an impractical comparison when considering the most common clinical scenario of an unwell, fasting patient presenting clinically with cholecystitis. Subsequent research has instead examined the utility of US in the critically ill patient cohort who present with features of cholecystitis, and report a highly variable sensitivity between 30% and 100%, with a more consistent specificity between 89% and 97% (excluding studies with < 20 participants) [18–20, 41–43].

The differences in reported sensitivity make it challenging to draw any sensible conclusions and indicate that there are likely unknown variables impacting recorded results. This may be differences in technician skill level, inconsistent diagnostic criteria (although Pelinka et al and Puc et al have consistent criteria and present vastly different sensitivities), insufficiently sized research cohorts and differences in patient cohort demographics. In any event, given the unclear sensitivity of the US, using clinical findings to guide the management of patients presenting with cholecystitis is crucial, regardless of negative findings. Alternatively, the consistently high specificity of US can reassure clinicians that a positive US diagnosis in the presence of symptoms is likely genuine.

#### Screening for AAC with the US

The value of screening critically ill patients with US for undetected AAC is minimal, indeed, the prevalence of abnormal US findings in critically ill patients who never suffer from AAC is surprisingly high. Research performed by Boland et al and Myrianthefs et al place the prevalence of at least one US finding suggestive of AAC in ICU patients between 47.2% and 85%, while between 30.2% and 57% have at least three. This is in stark contrast to the roughly 5% of patients who were actually diagnosed and treated surgically for AAC in both studies [21, 22]. One reason why the US has such low specificity in critically ill patients is that some signs, for example, hydrops and sludge, may occur normally in a patient fasting for prolonged periods. This is not to say that the US is not important in the diagnosis and management of AAC, but correlation with the clinical picture is critical for accurate interpretation.

#### Prognostic value of US in AAC

Researchers have examined the complication rate of AAC patients assessed with US, aiming to evaluate the prognostic value of US findings. One such study by Lin et al examined a cohort of 92 patients who had been admitted > 20 days and had clinical suspicion of AAC. They found a statistically significant link between ≥ 3 positive US findings and eventual complications linked to AAC, most notably perforation [36]. Thampy et al performed a similar study on 94 patients with a haematological malignancy and had a similar result. In both studies, the rate of complications after a positive US diagnosis was high, between 21% and 38.7%. In contrast, the complication rate for patients with a negative scan was zero [36, 44]. These results emphasise the importance of the US for the early treatment and prevention of severe complications related to AAC. In terms of US signs that are most pertinent, the presence of wall thickness ≥ 3.5 mm, pericholecystic fluid, hydrops (> 4 cm transverse), and echogenic sludge were all significantly associated with complications in AAC (*p* < 0.05) [36, 44]. On the other hand, not all literature on this topic confirms a correlation. Research from Joseph et al and Sosna et al observed patients with suspected AAC who underwent cholecystostomy, both found no statistically significant link between sonographic findings and outcomes [45, 46].

#### Serial US scanning in AAC

One key advantage of US is the ease of regular scans to monitor disease, and the literature suggests this holds considerable value in the diagnosis and management of AAC. For hospitalised patients with a clinical picture suggestive of cholecystitis, the rate of positive sonographic diagnosis one week after an initially negative test is between 39% and 58% [36, 44]. Evidently, regular scanning would be useful to catch any cases missed on an initial scan, particularly considering the low sensitivity of US. In Pelinka et al’s study, serial US examinations were used for patients who initially demonstrated signs of AAC to monitor progression or resolution and ensured a 100% specificity (proven on histology) for all patients who had a cholecystectomy [41]. This demonstrates the utility of serial US in preventing unnecessary procedures and maximising diagnostic accuracy.

### HIDA scan

HIDA scan, also known as radionuclide cholescintigraphy (RC), is a nuclear study which has been proposed as an adjunct to US in the diagnosis of AAC. Originally described in the late 1970s with the introduction of ^99m^Tc-labelled iminodiacetic acid, its diagnostic application is well defined in many biliary diseases, including ACC, biliary obstruction, biliary atresia, and biliary dyskinesia [47, 48]. Somewhat surprisingly, then, its usefulness in AAC is still debated. The following section discusses the evidence for its use in the diagnosis of AAC (Table 3).

#### Acquisition process for HIDA scans

**Fig. 2:**
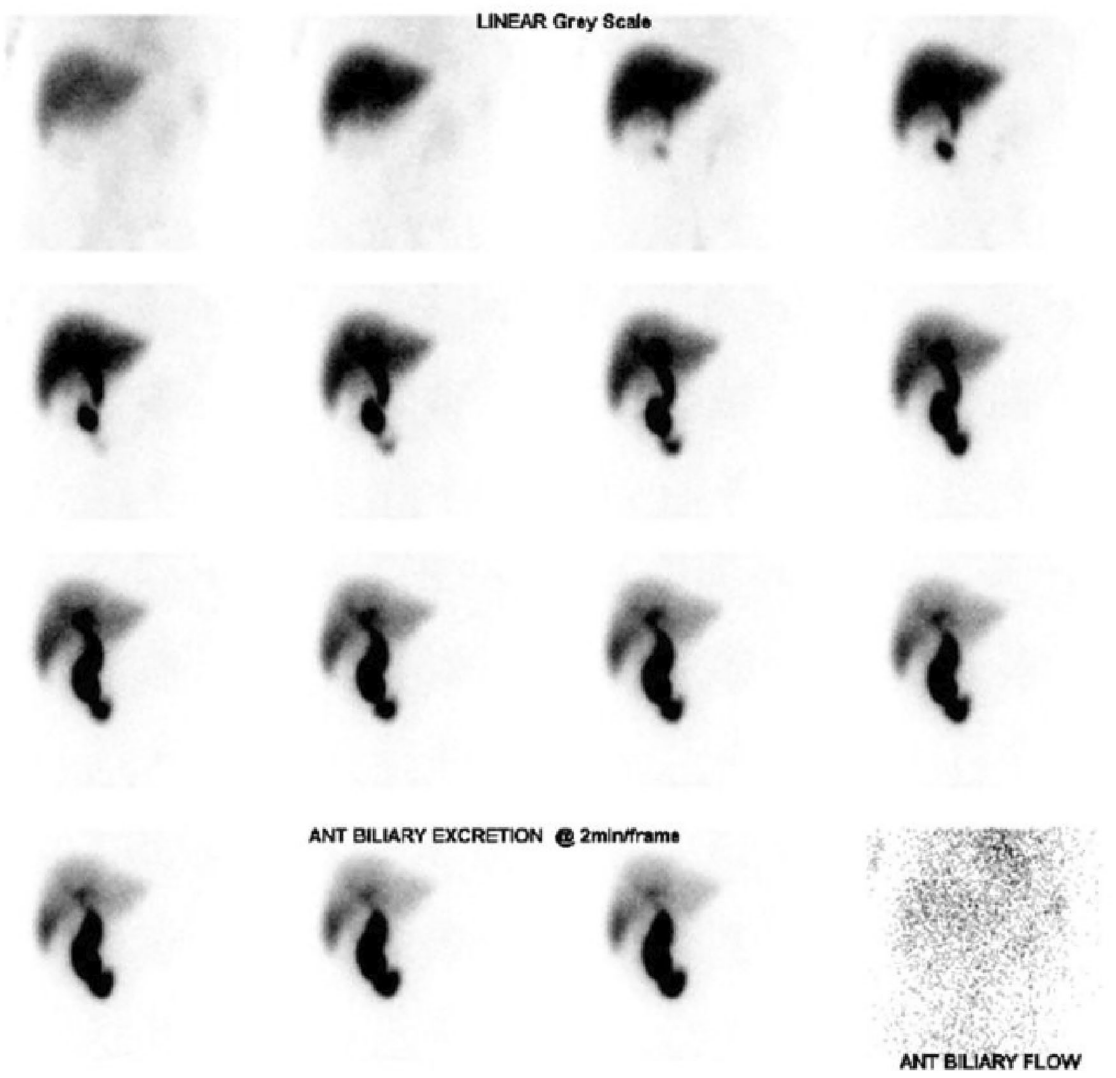
HIDA scan demonstrating failure of the gallbladder to fill with the radioactive tracer and possible medial deviation of the common bile duct, features which are consistent with a clinical picture of AAC. There is normal hepatic uptake of radiotracer activity and appropriate passage of activity from the biliary tree into the small intestine

**Fig. 3:**
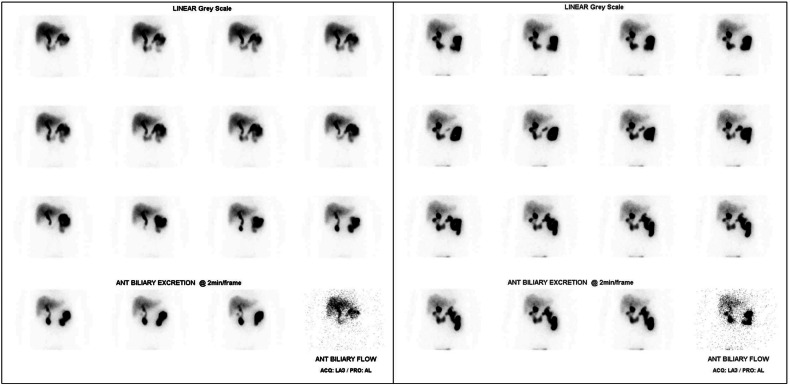
HIDA scan of a patient with suspected AAC, acquired over 90 min. Radiotracer activity and passage is seen through the liver, bile duct and duodenum, with no radiotracer activity in the gallbladder. The absence of radiotracer filling of the gallbladder in this context is compatible with AAC

In a HIDA scan, a radioactive tracer is administered intravenously and preferentially absorbed by the liver, where it is subsequently excreted within hepatic bile. In a healthy patient, some bile should be partitioned to the gallbladder, and therefore, if the gallbladder is not visualised within the first hour, the test can be considered diagnostic (Figs. 2 and 3) [49, 50]. The cause of a positive HIDA result in AAC is likely multifactorial. Increased wall oedema from inflammation due to tissue injury compresses the neck of the gallbladder, reducing inflow from the cystic duct and preventing uptake of hepatic bile. Additionally, reduced gallbladder contraction from either reduced cholinergic stimulation with prolonged fasting, or injury from infection or ischaemia results in a build-up of inspissated bile and prevents radiotracer uptake [2]. HIDA is additionally used to evaluate for biliary obstruction, in which the tracer will not be visualised in the duodenum, and chronic acalculous cholecystitis, where there is inadequate excretion of tracer from the gallbladder following administration of CKK [51, 52].

#### Diagnostic utility of HIDA in AAC

To date, the utility of HIDA scans for the diagnosis of AAC is not clearly established in the literature. Most papers exploring this issue are retrospective cohort studies, examining the ICU and trauma patient groups. Broadly, the sensitivity is reported between 38% and 100%, and the specificity is reported between 78% and 100% (excluding studies with < 20 participants) [18–20, 28, 42, 43, 53–55]. The variation in sensitivity is likely a consequence of the inconsistent criteria and augmentation techniques used for diagnosis. While the low specificity recorded in some studies can be explained by several factors, including gallbladder stasis from prolonged fasting, severe liver disease, chronic acalculous cholecystitis, analgesics, and severe illness. A significant disadvantage of HIDA is the lengthy scan acquisition time, at least an hour, which is often unsafe for the critically ill patient cohort who present with AAC.

#### Utility of HIDA augmentation techniques

In the context of AAC, there are two techniques which are used for scan augmentation, in an effort to provide more accurate results. Morphine may be administered to induce constriction of the sphincter of Oddi, a technique referred to as morphine cholescintigraphy (MC). The resulting increase in intraductal pressure should cause increased tracer uptake in the gallbladder, and if there is non-visualisation 30 min post morphine administration, it is considered a positive result. In lieu of morphine, performing a delayed scan 3–4 h post tracer administration is acceptable, although it may be challenging in the context of a critically ill patient.

Multiple studies have tested using both MC and a prolonged acquisition time to improve the accuracy of HIDA. Prévôt et al demonstrated an increase in specificity from 78% to 100% using MC compared to traditional HIDA, while Mariat et al also achieved a specificity of 100% using this technique [18, 19]. Increasing HIDA acquisition time also appears to improve specificity, and Mirvis et al achieved a specificity of 95% on their study of 42 patients, using an extended wait time of 6 h prior to labelling a positive result [43]. Unfortunately, it appears in both cases the improved specificity comes at the cost of reduced sensitivity, and the reported sensitivity of MC or a prolonged acquisition time is between 38% and 67% [18, 19, 43], compared to the sensitivity of between 95% and 100% seen in standard HIDA scans (for studies with greater than 20 participants) [20, 55]. Either way, while offering an improved specificity, both techniques appear to reduce sensitivity too substantially for consideration in AAC diagnosis.

### CT and MRI

**Fig. 4:**
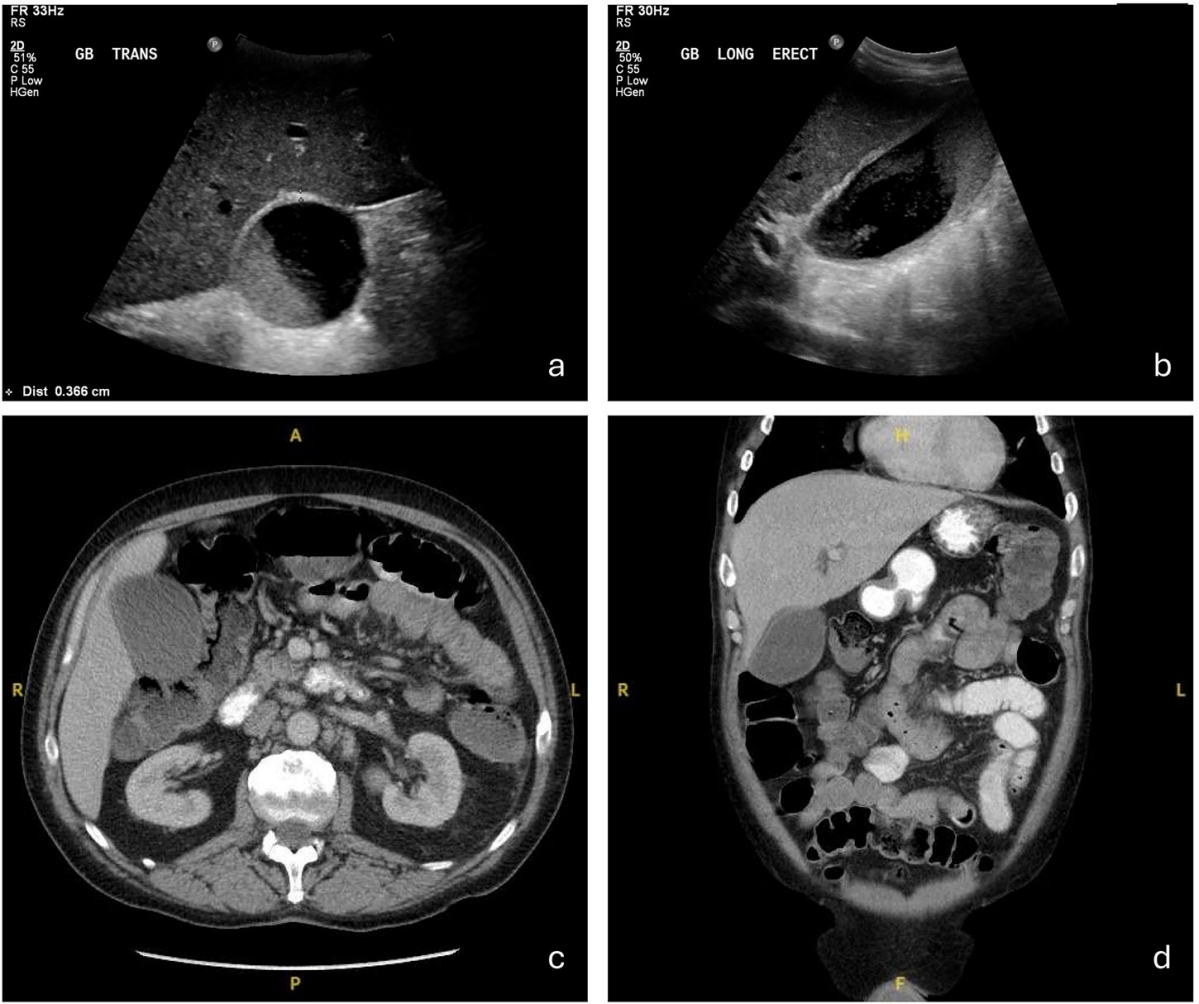
Fifty-nine-year-old male with suspected acalculous cholecystitis. He presented on day 10 post-chemotherapy with abdominal pain, rising inflammatory markers and a bilirubin of 60. Transverse (**a**) and longitudinal (**b**) views on US demonstrate gallbladder sludge and wall thickening of 3.6 mm. Axial and transverse planes on CT (**c**, **d**) show gallbladder distension, wall thickening, and no radiopaque stones. The patient subsequently underwent cholecystostomy, which was successful at preventing further complications

**Fig. 5:**
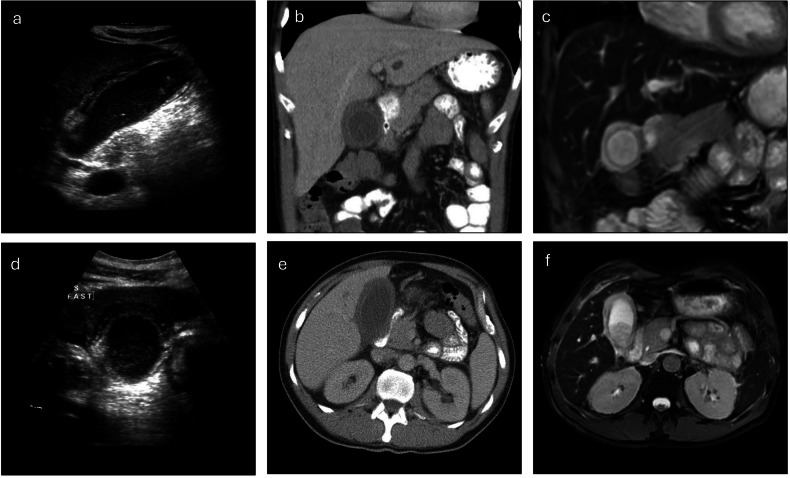
Fifty-three-year-old male with histologically proven acalculous cholecystitis managed with cholecystectomy. Longitudinal (**a**) and transverse (**d**) US views of the gallbladder demonstrate an oedematous gallbladder with wall thickening measuring 4.5 mm, biliary sludge, and no identifiable gallstones. Contrast-enhanced coronal (**b**) and axial (**e**) CT images show a persistently distended gallbladder with diffuse mural thickening and no radiopaque gallstones identified. Coronal (**c**) and axial (**f**) T2-weighted fat-saturated MR images reveal persistent gallbladder wall thickening and inspissated bile. No gallstones are visualised

**Fig. 6:**
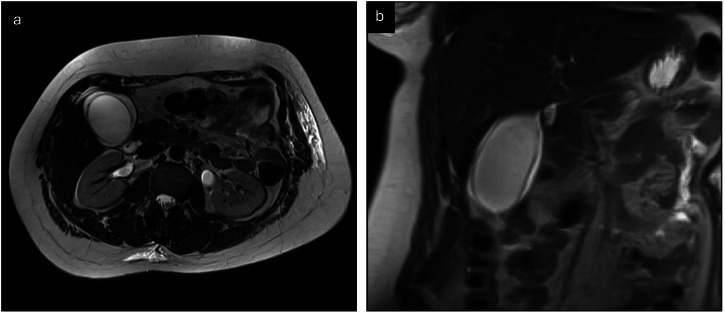
Thirty-five-year-old female with histologically proven AAC. Axial (**a**) and coronal (**b**) T2-weighted MR images demonstrate a distended gallbladder with oedematous wall thickening. There is no evidence of inspissated bile or stones

CT and MRI have been explored as potential diagnostic tools for AAC (Figs. 4–6). Unfortunately, CT has been shown to provide little benefit over US, and the requirement for transport to a scanner limits access for the critically ill patient. Mirvis et al defined a CT diagnosis criterion which achieved an impressive sensitivity and specificity of 100% on their cohort of 15 patients, however, further research has put the sensitivity of this criterion for AAC diagnosis closer to 25% (Table 4) [31, 37, 43, 56]. Additionally, similarly to the US, CT screening for AAC in ICU patients without clinical suspicion is not useful, as nonspecific abnormalities are seen in 83%, yet only a small fraction truly develop AAC [21, 22, 31]. Evidently, the diagnostic value of CT over US for AAC diagnosis is minimal, however, it still holds utility in ruling out alternative abdominal pathology. On the other hand, there is no specific research examining the diagnostic benefit of MRI in acalculous cholecystitis. MRI (i.e. magnetic resonance cholangiopancreatograph (MRCP) protocol) holds significant utility in the diagnosis of acute cholecystitis as a whole, and a meta-analysis in 2012 demonstrated MRI has a sensitivity of 85% and a specificity of 81%, while Kaura et al demonstrated T2 weighted MRI has a superior specificity than CT for the detection of cholecystitis [30, 57, 58]. In addition, MRI is useful for the detection of common AAC complications, including gangrenous cholecystitis or perforation, and provides an excellent view of the biliary tree, which is beneficial for surgical planning. Considering this, MRI is likely a useful modality for the evaluation of AAC, unfortunately, the somewhat arduous and relatively prolonged image acquisition time limits accessibility for critically ill patients [59].

### Utility of combined imaging approaches

An important consideration for clinicians managing suspected AAC is the benefit of a multimodal imaging approach to improve diagnostic accuracy, however, this has received little attention in the literature. There are several examples of AAC case studies—proven on histology or managed effectively with a cholecystostomy—which showed equivocal US and CT scans but were subsequently diagnosed on HIDA or MRCP [60–64]. However, the only cohort study which explored this relationship was performed by Mariat et al, and employed a Bayesian method to investigate the utility of combining HIDA and US. It found the probability of having AAC decreased from 86% with a positive US to 66% if the subsequent HIDA was negative, while a positive HIDA was 100% specific for AAC in this study, regardless of the US result [19]. This led the authors to recommend adjuvant HIDA in the context of an equivocal US result. Nevertheless, given this is the only study to examine the combined modality efficacy besides individual case studies, the utility of combing HIDA and US remains unclear.

## Implications for practice

Given inconsistencies between studies, both in terms of methodology and results, it is challenging to draw conclusions, however, our advice is outlined in the following (Table 5). US is a crucial diagnostic tool due to its accessibility and specificity, particularly when the clinical picture strongly suggests AAC, although caution should be taken with screening asymptomatic patients, as the rate of false positives is high. Its low sensitivity limits its ability to rule out the condition, emphasising the importance of corroborating imaging findings with clinical presentation. Serial scanning appears to enhance diagnostic sensitivity and may aid in monitoring disease progression. CT, while offering little additional benefit over US for diagnosing AAC directly, can be valuable in screening for alternative abdominal pathology. MRI may have potential in AAC diagnosis due to its proven utility in acute cholecystitis; however, its role is constrained by logistical limitations, similar to those of HIDA scans. While HIDA scans provide high specificity, their limited sensitivity, coupled with the significant time and resources required for acquisition, restricts their utility, particularly in critically ill patients. Both modalities should be considered on a case-by-case basis, as they are unlikely to significantly alter management in most scenarios. Nevertheless, there should be a low threshold for intervention if AAC is suspected, as it is crucial to preventing complications. The TG18 recommends laparoscopic cholecystectomy in the first instance, or in patients unfit for surgery, percutaneous cholecystostomy as a temporising measure [65–68].

**Table 5:** Reported sensitivity, specificity, advantages and disadvantages of each imaging modality for AAC diagnosis

	Sensitivity	Specificity	Advantages	Limitations
US	30–100%	89–97%	• Can be performed at the patient's bedside • No radiation concerns with serial scanning • Relatively quick scan to acquire	• The skill level of the sonographer will impact the scan
HIDA	38–100%	78–100%	N/A	• Time-consuming to acquire • Requires transport to the machine
CT	22%	Not tested	• Useful to rule out other intra-abdominal pathology	• Requires transport to the machine • Radiation exposure
MRI	Not tested	Not tested	• Useful for the characterisation of complications of AAC • The entire biliary tree is assessed	• Time-consuming to acquire • Requires transport to the machine

Percentages are for patients with clinical suspicion of AAC

## Future directions

Looking forward, advances in both artificial intelligence (AI) and imaging technology are likely to aid the radiological diagnosis of AAC. AI models, while not specifically tested for AAC, have been utilised in the diagnosis of other gallbladder diseases, including acute cholecystitis, cholelithiasis, gallbladder polyps and gallbladder malignancy [69–71]. In one study, a computer vision model achieved an impressive diagnostic sensitivity of 87% and specificity of 89% for acute cholecystitis on US captured still images [72]. While promising, a significant challenge with this approach is the paucity of imaging examples for AAC, limiting the training data available for model development. This issue is not unique to AAC, and there has been interest in the use of large generalisable foundation models which can be optimised for few-shot diagnosis on limited training samples, which in future may be used for computer-aided diagnosis of AAC [73].

Advances in imaging technology may additionally aid AAC diagnosis. Dual energy CT is widely used to identify radio-opaque gallstones on CT in calculous cholecystitis [74], and the development of multispectral CT may further its applications through the acquisition of higher resolution images and identification of tissue not previously differentiable on traditional CT [75]. There is evidence that this technology is useful for the detection of important cholecystitis features such as mural hyperaemia and hypervascularity of adjacent liver parenchyma [76]. Additionally, there has been interest in using diffusion-weighted MRI sequences to better characterise gallbladder wall thickening and inflammation, predict challenging cholecystectomies, and differentiate between chronic and acute cholecystitis [77, 78]. Nevertheless, the application of these technologies to AAC remains theoretical and has not been tested.

## Limitations of the review

Despite offering a comprehensive synthesis of imaging in AAC, several limitations must be acknowledged. The use of a single reviewer for study selection may introduce bias; however, this was mitigated by consulting a second reviewer for articles where inclusion was unclear. Furthermore, direct comparisons between studies were not possible as there was significant heterogeneity in diagnostic criteria. Additionally, strong conclusions about causality could not be drawn from most included studies due to their retrospective nature. Potential publication bias may also be present, as negative findings are often underreported.

## Conclusion

In summary, while radiological assessment holds definite utility in the management of suspected AAC, further research is required to properly define its role. US remains the primary imaging modality in the diagnosis of AAC, largely due to its accessibility, bedside applicability, and high specificity. However, variability in sensitivity across studies suggests that reliance on the US alone may be insufficient, particularly in critically ill populations. Serial US imaging has emerged as a promising approach to improve diagnostic sensitivity, though standardised protocols for its application are lacking in the literature. While HIDA scans have been explored as a complementary modality to US, particularly in cases with inconclusive US findings, their limited sensitivity and logistical challenges restrict their widespread use. CT, although valuable for identifying alternative intra-abdominal pathology, offers a limited diagnostic advantage over US for AAC. MRI holds established utility in many biliary conditions; however, it lacks robust evidence to support its routine use in AAC diagnosis. Currently, there is a lack of prospective studies, and no standardised diagnostic criteria across imaging modalities, which complicates the comparison and interpretation of study findings. Future research should focus on well-designed prospective studies to establish the diagnostic performance of both individual and multimodal imaging strategies in AAC, as well as leveraging AI and novel CT and MRI technologies to improve diagnostic accuracy. Furthermore, standardising imaging criteria and protocols across institutions will be essential to improving diagnostic consistency and patient outcomes in this under-researched area.

## Supplementary information

ELECTRONIC SUPPLEMENTARY MATERIAL

## Abbreviations

AAC: Acute acalculous cholecystitis
ACC: Acute calculous cholecystitis
AI: Artificial intelligence
CT: Computed tomography
HIDA: Hepatobiliary iminodiacetic acid
ICU: Intensive care unit
MC: Morphine cholescintigraphy
MRCP: Magnetic resonance cholangiopancreatography
MRI: Magnetic resonance imaging
PRISMA-ScR: Preferred reporting items for systematic reviews and meta-analyses extension for scoping reviews
RC: Radionuclide cholescintigraphy
TG18: Tokyo Guidelines 2018
US: Ultrasound
