# Supplementary material for: What imaging modalities should be considered in suspected acute acalculous cholecystitis? A review of the evidence
Source: Insights Imaging. 2025 Nov 19;16:257. doi: 10.1186/s13244-025-02106-2 (PMC12630445; doi:10.1186/s13244-025-02106-2)

Supplementary Materials

1.0 Search Queries

1.1 PubMED search strategy

<u>The simplified search query is stated below:</u>

((Acalculous cholecystitis) AND ((ultrasound) OR (sonography) OR (computed tomography) OR (CT) OR (magnetic resonance imaging) OR (MRI) OR (Magnetic resonance cholangiopancreatography) OR (MRCP) OR (hepatobiliary scintigraphy) OR (HIDA) OR (cholescintigraphy) OR (imaging) OR (scan))) NOT (Case Reports[Publication Type])

Filters: English, Humans, from 1980/1/1 - 2025/1/1

Number of results: 321

<u>The detailed search query is also outlined:</u>

((("acalculous cholecystitis"[MeSH Terms] OR ("acalculous"[All Fields] AND "cholecystitis"[All Fields]) OR "acalculous cholecystitis"[All Fields]) AND ("diagnostic imaging"[MeSH Subheading] OR ("diagnostic"[All Fields] AND "imaging"[All Fields]) OR "diagnostic imaging"[All Fields] OR "ultrasound"[All Fields] OR "ultrasonography"[MeSH Terms] OR "ultrasonography"[All Fields] OR "ultrasonics"[MeSH Terms] OR "ultrasonics"[All Fields] OR "ultrasounds"[All Fields] OR "ultrasound s"[All Fields] OR ("sonographies"[All Fields] OR "ultrasonography"[MeSH Terms] OR "ultrasonography"[All Fields] OR "sonography"[All Fields]) OR ("tomography, x ray computed"[MeSH Terms] OR ("tomography"[All Fields] AND "x ray"[All Fields] AND "computed"[All Fields]) OR "x-ray computed tomography"[All Fields] OR ("computed"[All Fields] AND "tomography"[All Fields]) OR "computed tomography"[All Fields]) OR ("j comput tomogr"[Journal] OR "commun theory"[Journal] OR "child teenagers"[Journal] OR "cancer ther"[Journal] OR "ct"[All Fields]) OR ("magnetic resonance imaging"[MeSH Terms] OR ("magnetic"[All Fields] AND "resonance"[All Fields] AND "imaging"[All Fields]) OR "magnetic resonance imaging"[All Fields]) OR ("magnetic resonance imaging"[MeSH Terms] OR ("magnetic"[All Fields] AND "resonance"[All Fields] AND "imaging"[All Fields]) OR "magnetic resonance imaging"[All Fields] OR "mri"[All Fields]) OR ("cholangiopancreatography, magnetic resonance"[MeSH Terms] OR ("cholangiopancreatography"[All Fields] AND "magnetic"[All Fields] AND "resonance"[All Fields]) OR "magnetic resonance cholangiopancreatography"[All Fields] OR ("magnetic"[All Fields] AND "resonance"[All Fields] AND "cholangiopancreatography"[All Fields])) OR "MRCP"[All Fields] OR ("hepatobiliary"[All Fields] AND ("radionuclide imaging"[MeSH Terms] OR ("radionuclide"[All Fields] AND "imaging"[All Fields]) OR "radionuclide imaging"[All Fields] OR "scintigraphies"[All Fields] OR "scintigraphy"[All Fields])) OR ("lidofenin"[Supplementary Concept] OR "lidofenin"[All Fields] OR "hida"[All Fields]) OR "cholescintigraphy"[All Fields] OR ("image"[All Fields] OR "image s"[All Fields] OR "imaged"[All Fields] OR "imager"[All Fields] OR "imager s"[All Fields] OR "imagers"[All Fields] OR "images"[All Fields] OR "imaging"[All Fields] OR "imaging s"[All Fields] OR "imagings"[All Fields]) OR ("radionuclide imaging"[MeSH Terms] OR ("radionuclide"[All Fields] AND "imaging"[All Fields]) OR "radionuclide imaging"[All Fields] OR "scan"[All Fields]))) NOT "case reports"[Publication Type]) AND ((humans[Filter]) AND (1980/1/1:2025/1/1[pdat]) AND (english[Filter]))

Translations

Acalculous cholecystitis: "acalculous cholecystitis"[MeSH Terms] OR ("acalculous"[All Fields] AND "cholecystitis"[All Fields]) OR "acalculous cholecystitis"[All Fields]

ultrasound: "diagnostic imaging"[Subheading] OR ("diagnostic"[All Fields] AND "imaging"[All Fields]) OR "diagnostic imaging"[All Fields] OR "ultrasound"[All Fields] OR "ultrasonography"[MeSH Terms] OR "ultrasonography"[All Fields] OR "ultrasonics"[MeSH Terms] OR "ultrasonics"[All Fields] OR "ultrasounds"[All Fields] OR "ultrasound's"[All Fields]

sonography: "sonographies"[All Fields] OR "ultrasonography"[MeSH Terms] OR "ultrasonography"[All Fields] OR "sonography"[All Fields]

computed tomography: "tomography, x-ray computed"[MeSH Terms] OR ("tomography"[All Fields] AND "x-ray"[All Fields] AND "computed"[All Fields]) OR "x-ray computed tomography"[All Fields] OR ("computed"[All Fields] AND "tomography"[All Fields]) OR "computed tomography"[All Fields]

CT: "J Comput Tomogr"[Journal:__jid7805373] OR "Commun Theory"[Journal:__jid9886794] OR "Child Teenagers"[Journal:__jid101773087] OR "Cancer Ther"[Journal:__jid101174596] OR "ct"[All Fields]

magnetic resonance imaging: "magnetic resonance imaging"[MeSH Terms] OR ("magnetic"[All Fields] AND "resonance"[All Fields] AND "imaging"[All Fields]) OR "magnetic resonance imaging"[All Fields]

MRI: "magnetic resonance imaging"[MeSH Terms] OR ("magnetic"[All Fields] AND "resonance"[All Fields] AND "imaging"[All Fields]) OR "magnetic resonance imaging"[All Fields] OR "mri"[All Fields]

Magnetic resonance cholangiopancreatography: "cholangiopancreatography, magnetic resonance"[MeSH Terms] OR ("cholangiopancreatography"[All Fields] AND "magnetic"[All Fields] AND "resonance"[All Fields]) OR "magnetic resonance cholangiopancreatography"[All Fields] OR ("magnetic"[All Fields] AND "resonance"[All Fields] AND "cholangiopancreatography"[All Fields])

scintigraphy: "radionuclide imaging"[MeSH Terms] OR ("radionuclide"[All Fields] AND "imaging"[All Fields]) OR "radionuclide imaging"[All Fields] OR "scintigraphies"[All Fields] OR "scintigraphy"[All Fields]

HIDA: "lidofenin"[Supplementary Concept] OR "lidofenin"[All Fields] OR "hida"[All Fields]

imaging: "image"[All Fields] OR "image's"[All Fields] OR "imaged"[All Fields] OR "imager"[All Fields] OR "imager's"[All Fields] OR "imagers"[All Fields] OR "images"[All Fields] OR "imaging"[All Fields] OR "imaging's"[All Fields] OR "imagings"[All Fields]

scan: "radionuclide imaging"[MeSH Terms] OR ("radionuclide"[All Fields] AND "imaging"[All Fields]) OR "radionuclide imaging"[All Fields] OR "scan"[All Fields]

1.2 Ovid MEDLINE(R) ALL Search Query

The search query is outlined below:

1. Ultrasound.mp. [mp=title, book title, abstract, original title, name of substance word, subject heading word, floating sub-heading word, keyword heading word, organism supplementary concept word, protocol supplementary concept word, rare disease supplementary concept

word, unique identifier, synonyms, population supplementary concept word, anatomy supplementary concept word]

2. sonograph*.mp. [mp=title, book title, abstract, original title, name of substance word, subject heading word, floating sub-heading word, keyword heading word, organism supplementary concept word, protocol supplementary concept word, rare disease supplementary concept word, unique identifier, synonyms, population supplementary concept word, anatomy supplementary concept word]
3. computed tomography.mp. [mp=title, book title, abstract, original title, name of substance word, subject heading word, floating sub-heading word, keyword heading word, organism supplementary concept word, protocol supplementary concept word, rare disease supplementary concept word, unique identifier, synonyms, population supplementary concept word, anatomy supplementary concept word]
4. CT.mp. [mp=title, book title, abstract, original title, name of substance word, subject heading word, floating sub-heading word, keyword heading word, organism supplementary concept word, protocol supplementary concept word, rare disease supplementary concept word, unique identifier, synonyms, population supplementary concept word, anatomy supplementary concept word]
5. magnetic resonance imaging.mp. [mp=title, book title, abstract, original title, name of substance word, subject heading word, floating sub-heading word, keyword heading word, organism supplementary concept word, protocol supplementary concept word, rare disease supplementary concept word, unique identifier, synonyms, population supplementary concept word, anatomy supplementary concept word]
6. MRI.mp. [mp=title, book title, abstract, original title, name of substance word, subject heading word, floating sub-heading word, keyword heading word, organism supplementary concept word, protocol supplementary concept word, rare disease supplementary concept word, unique identifier, synonyms, population supplementary concept word, anatomy supplementary concept word]
7. Magnetic Resonance Cholangiopancreatography.mp. [mp=title, book title, abstract, original title, name of substance word, subject heading word, floating sub-heading word, keyword heading word, organism supplementary concept word, protocol supplementary concept word, rare disease supplementary concept word, unique identifier, synonyms, population supplementary concept word, anatomy supplementary concept word]
8. MRCP.mp. [mp=title, book title, abstract, original title, name of substance word, subject heading word, floating sub-heading word, keyword heading word, organism supplementary concept word, protocol supplementary concept word, rare disease supplementary concept word, unique identifier, synonyms, population supplementary concept word, anatomy supplementary concept word]
9. Hepatobiliary scintigraphy.mp. [mp=title, book title, abstract, original title, name of substance word, subject heading word, floating sub-heading word, keyword heading word, organism supplementary concept word, protocol supplementary concept word, rare disease supplementary concept word, unique identifier, synonyms, population supplementary concept word, anatomy supplementary concept word]
10. HIDA.mp. [mp=title, book title, abstract, original title, name of substance word, subject heading word, floating sub-heading word, keyword heading word, organism supplementary concept word, protocol supplementary concept word, rare disease supplementary concept word, unique identifier, synonyms, population supplementary concept word, anatomy supplementary concept word]

11. Cholescintigraphy.mp. [mp=title, book title, abstract, original title, name of substance word, subject heading word, floating sub-heading word, keyword heading word, organism supplementary concept word, protocol supplementary concept word, rare disease supplementary concept word, unique identifier, synonyms, population supplementary concept word, anatomy supplementary concept word]
12. imag*.mp. [mp=title, book title, abstract, original title, name of substance word, subject heading word, floating sub-heading word, keyword heading word, organism supplementary concept word, protocol supplementary concept word, rare disease supplementary concept word, unique identifier, synonyms, population supplementary concept word, anatomy supplementary concept word]
13. scan*.mp. [mp=title, book title, abstract, original title, name of substance word, subject heading word, floating sub-heading word, keyword heading word, organism supplementary concept word, protocol supplementary concept word, rare disease supplementary concept word, unique identifier, synonyms, population supplementary concept word, anatomy supplementary concept word]
14. 1 or 2 or 3 or 4 or 5 or 6 or 7 or 8 or 9 or 10 or 11 or 12 or 13
15. acalculous.mp. [mp=title, book title, abstract, original title, name of substance word, subject heading word, floating sub-heading word, keyword heading word, organism supplementary concept word, protocol supplementary concept word, rare disease supplementary concept word, unique identifier, synonyms, population supplementary concept word, anatomy supplementary concept word]
16. cholecystitis.mp. [mp=title, book title, abstract, original title, name of substance word, subject heading word, floating sub-heading word, keyword heading word, organism supplementary concept word, protocol supplementary concept word, rare disease supplementary concept word, unique identifier, synonyms, population supplementary concept word, anatomy supplementary concept word]
17. 15 and 16
18. 14 and 17
19. Case Reports/
20. 18 not 19
21. limit 20 to (english language and humans and yr="1980 - 2025")

Number of results: 305

2.0 Scoping review methods

This scoping review was conducted by a single reviewer (B.P.), who was responsible for identifying, screening, and selecting studies. During the initial screening phase, the reviewer independently assessed all retrieved articles based on their title and abstract, using predefined eligibility criteria, excluding studies that did not meet the inclusion criteria. Articles that passed initial screening then underwent a full-text review, where they were evaluated for relevance, methodological quality, and clarity in defining AAC diagnostic criteria. Studies deemed irrelevant or methodologically weak were excluded, with reasons for exclusion documented at the full-text review stage. To enhance the reliability of the selection process, any uncertain cases were discussed with a second reviewer, ensuring a more robust decision-making process for borderline articles. A PRISMA flow diagram

illustrates the study selection process, including the reasons for exclusion at the full-text review stage. For each included study, the design, population characteristics (sample size, patient demographics, ICU vs. trauma cohort), imaging modalities evaluated (US, CT, MRI/MRCP, HIDA scan), diagnostic criteria used for diagnosis and diagnostic accuracy metrics (sensitivity, specificity, and overall accuracy) were recorded. Given the significant heterogeneity in study designs, methodologies, and definitions of AAC, a meta-analysis was not performed. Instead, results were synthesised narratively, categorised by imaging modality, and summarised in comparative tables to provide a structured overview of findings. This scoping review followed the Preferred Reporting Items for Systematic Reviews and Meta-Analyses extension for Scoping Reviews (PRISMA-ScR) guidelines to identify relevant studies. The eligibility criteria for study selection are outlined below. While protocol registration is not mandatory for scoping reviews, we preregistered and published our protocol on the Open Science Framework (OSF) to enhance transparency and reproducibility. The registered protocol is publicly available at https://doi.org/10.17605/OSF.IO/4R3XP.

Eligibility criteria used for study selection in the scoping review

Criteria	Inclusion Criteria	Exclusion Criteria
Population	- Human patients diagnosed with AAC, either clinically, radiologically, or histopathologically	- Studies focusing on acute calculous cholecystitis (ACC) or chronic acalculous cholecystitis (CAC) without specific mention of AAC<br>- Screening studies, for example methodology which required imaging of an entire cohort regardless of symptoms or clinical concern
Interventions	- Studies that evaluated the use of US, CT, MRI/MRCP, or HIDA scans in diagnosing AAC	- Studies with insufficient data on imaging performance or sample sizes of less than ten patients, unless they provided novel insights
Outcomes	- Studies that reported on diagnostic accuracy metrics (e.g., sensitivity, specificity, predictive values) or discussed imaging's impact on clinical decision-making	- Studies which did not use histological confirmation for AAC diagnosis
Publication type and language	- Studies published in English between 1980 until 2025<br>- Original research articles, retrospective and prospective cohort studies, case control studies, systematic reviews and	- Animal studies or research that lacked direct clinical correlation<br>- Case reports, conference abstracts, grey literature, and non-peer-reviewed studies

	meta-analyses	

PRIMSA flow diagram outlining the search procedure

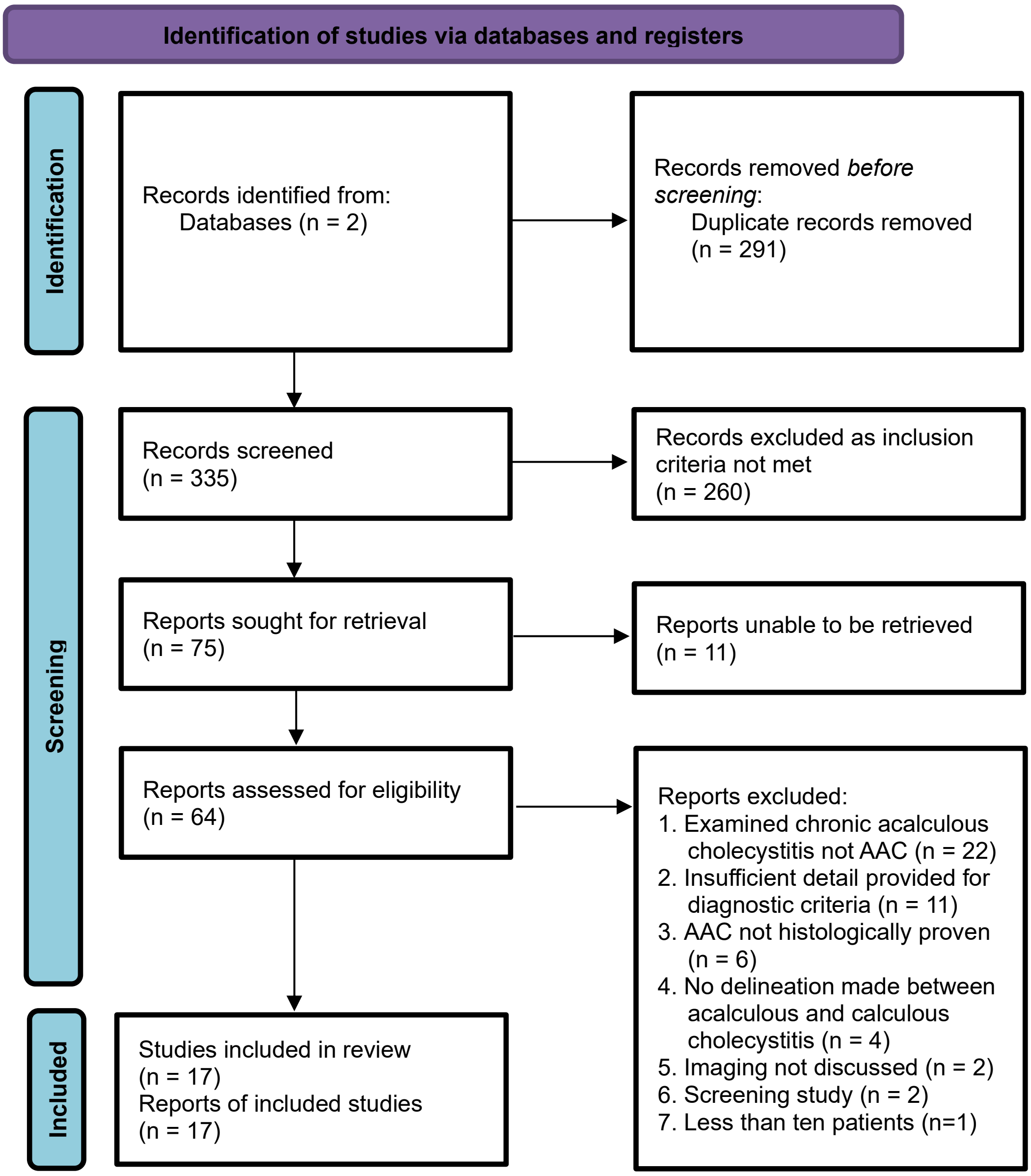

Supplement: Supplementary file 1 — ELECTRONIC SUPPLEMENTARY MATERIAL [file 13244_2025_2106_MOESM1_ESM.pdf]
